# Some facts and thoughts: p73 as a tumor suppressor gene in the network of tumor suppressors

**DOI:** 10.1186/1476-4598-6-27

**Published:** 2007-04-03

**Authors:** Lakshmanane Boominathan

**Affiliations:** 1 49, Nattar main street, Murungapakkam, Pondicherry-4, India

## Abstract

The question of whether p73 is a tumor suppressor gene, is not yet answered with full confidence. The lack of spontaneous tumor formation in p73 null mice and infrequent p73 mutations seen in a variety of cancers analyzed would straightaway negate its role as a primary tumor suppressor gene. However, accumulating evidence suggest that p73 gene and its target genes are hypermethylated in the cancer of lymphoid origin. Here I discuss some facts and thoughts that support the idea that p73 could still be a tumor suppressor gene. The tumor suppressor network in which p73 appears to be a participant involves E2F1, JunB, INK4a/p16, ARF/p19, p57kip2 and BRCA1. Knock out of each gene in E2F-1-p73-JunB-p16INK4a network of tumor suppressor proteins result in lymphoma/leukemia formation. Further, I tried to explain why lymphomas are not seen in p73 null mice and why p73 gene is not prone to frequent mutation.

## Background

p73 is a structural and functional homologue of the tumor suppressor, p53. Knock out studies suggest that p73 null mice do not produce spontaneous tumor formation even after two years. However, it appears that p73 shares the properties of the tumor suppressor p53. It functions more like its counterpart, p53, when it is overexpressed, it promotes growth inhibition or apoptosis.

## Evidence in favor of p73 as a main player in the network of tumor suppressors

**Figure 1:**
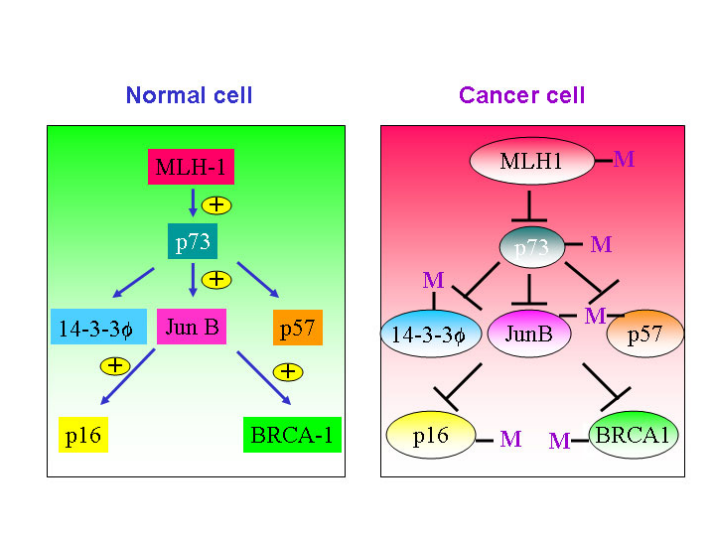
Methylation mechanism is evident in cancer cells but not in normal cells.

**Figure 2:**
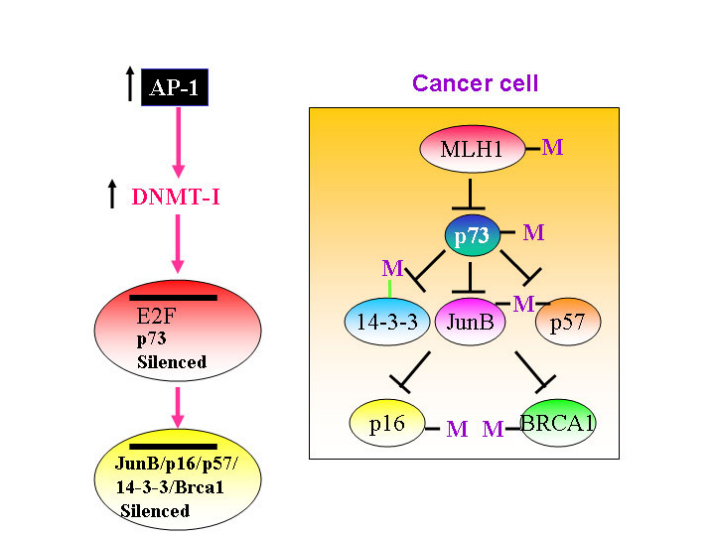
Increased AP-1 activity will upregulate DNMT-1 levels and thereby silence E2F-1, p73, JunB, p16, p57, 14-3-3 and BRCA-1 expression.

1. The p73 gene was methylated in 94% cases of natural killer cell lymphomas, a frequency that is the highest known for any human malignancy[1]. In the NK cell lymphoma line NK92, p73 was also completely methylated(figure 1), and the p73 transcript was correspondingly not detectable by quantitative polymerase chain reaction [2,3]. Treatment of the cell line with 5-azacytidine, a demethylation reagent, led to demethylation of the p73 promoter and reinduction of p73 gene expression. These results suggest the following: (a)Promoter CpG methylation might be an important mechanism in suppressing p73 gene expression in NK cells. (b) p73 can be a tumor suppressor in NK cells.

2. Epigenetic silencing mechanism has been shown to attenuate the expression of p73 in lymphoid cells but not in other tumors. p73 mutations seem to be infrequent, but some tumors do show LOH (20–60%). Interestingly, lymphoid tumors are not prone to LOH[4]. Genomic structure analysis of p73 promoter has found regions enriched in CpG islands, unlike p53 gene promoter. This could be one of the reasons why p73 is more prone to DNA methylation mechanisms, unlike p53. Aberrant methylation of p73 promoter has been detected in leukemia and lymphoma but not in blood cells(figure 1), supporting the possibility that appropriate methylation status of the CpG islands in the promoter region may play a crucial role in the downregulation of p73 gene expression. Unlike the p53 gene promoter, the human p73 gene promoter contained a putative TATA-box, and did not exhibit any extended homology to the p53 gene. Two CpG islands were located in the 5' upstream region. Transient transfection assays using progressive truncations of the p73 promoter showed that deletion from -119 to +19 relative to exon 1 resulted in a 13- to 20-fold reduction in the p73 promoter activity, suggesting that the elements for basal promoter activity exist in this region, where in putative Sp1, AP-2 and Egr-1, 2, 3 sites are located and CpG dinucleotides are especially concentrated[5].

3. It has been shown recently that promoter methylation of at least one tumor suppressor gene was present in 26/28 (92%) of the intraductal papillary mucinous neoplasms (IPMNs)[6]. The cell cycle control genes, p16 and p73, were methylated frequently (>50%) in both non-invasive and invasive tumors[7]. Pancreatic cancer is particularly resistant to apoptosis induced by antineoplastic agents, which is partly attributable to the lack of functional p53. However, it has been shown that E2F1 in combination with the most clinically efficient drug, gemcitabine, resulted in a strong induction of apoptosis independent of functional p53, whereas the effect of either therapy alone varied between different cell lines. Intratumoral injection of a helper-dependent adenovirus vector expressing E2F1 plus drug treatment resulted in a significant reduction of tumor volume[6]. The therapeutic effect is directly correlated with the induction of the p53 homologue p73, suggesting that the recently discovered E2F1/p73 pathway plays a critical role in cancer therapy.

4. The fact that *MLH-/- *fibroblasts do not induce p73 in response to cisplatin treatment indicate that MLH-1 is upstream to p73 and it is a participant in the DNA repair pathway [8,9]. MLH-1 has been shown to be hypermethylated in several tumors including gastric and hemotopoietic origin. If MLH is an upstream regulator of p73 in tumors (in which MLH is hypermethylated), then p73 may not be activated in these tumors in response to aberrant DNA damage signal(figure 1). If an upstream regulator of a tumor suppressor is defective, then it is obvious that the whole pathway leading to the induction of tumor suppressor gene will be defective. For example, tumors in which Chk2, an upstream regulator of p53, is mutated, p53 mutations are infrequent.

5. JunB has been shown to be a target gene of p73-α[10]. JunB is a negative regulator of proliferation and it has been shown to induce the transcription of p16 in fibroblasts[11]. Jun B has also been shown to potentiate the function of BRCA1, which is not only expressed in mammary and ovary glands but also in T-cells as well[12] (figure 1). Jun B transgenic mice lost its expression in myeloid lineage resulting in chronic myeloid leukemia, indicating that JunB acts as a tumor suppressor gene in myeloid cells[13]. Silencing of p73 expression by hypermethylation or by any other mechanisms leading to the poor expression of the p73 protein could impair the expression of Jun B in myeloid cells. JunB serves as a causative factor in the development of chronic myeloid leukemia. Both JunB and Jun D are located on human chromosome 19 p13.2, a region that may be involved in chromosomal translocation in acute lymphocytic leukemia (ALL), acute nonlymphocytic leukemia (ANLL) and malignant melanoma (MEL).

6. p16 has been shown to function as a tumor suppressor gene. Further, it has been shown that JunB, a transcriptional target of p73, potentiates the expression of p16[11] (figure 1). The existence of this pathway is evident from the fact that overexpresssion of p16INK4a correlates with high expression of p73[14]. Furthermore, In a group of 16 patients with acute lymphoplastic leukemia(ALL), p16 was methylated in 11.7% of patients, and p73 was methylated in 17.6% of patients. This data highlights the importance of inactivation of p73-JunB-p16 pathway in lymphoplastic leukemia.

7. p57Kip2 is yet another player in the tumor suppressor network. p73-beta has been shown to transactivate p57Kip2 expression[14]. Further it has been shown that p57Kip2 promotes growth arrest in T-cells[15]. p57Kip1 promoter is hypermethylated in a wide variety of lymphoid malignancies. p57KIP2 was found to be frequently methylated (50%) in acute lymphocytic leukemia (ALL)-derived cell lines[16].

8. 14-3-3 sigma has been shown to be a transcriptional target of p53 and p73 and regulates G2/M phase[17]. It has been shown that diminished or decreased expression of 14-3-3 sigma results in increased chemoresistance of cancer cells[18]. Further, 14-3-3 sigma blocks Akt-mediated degradation of p27Kip2 and thereby enhancing the stability of the cell cycle inhibitor p27. Thus, p73, by potentiating the expression of 14-3-3 sigma, increases the chemosensitvity of drug resistant cancer cells[19]. 14-3-3 is hypermethylated in breast cancer(24 of 25 carcinomas (96%), 15 of 18 (83%) of ductal carcinoma), 89% (17/19) of the HCC and 26 of 60 (43%) of gastric cancers [20-23].

9. BRCA1 appears to be a selective co-activator of 14-3-3 sigma gene transcription[24].

10. Both low E2F-1 expression and p16INK4A inactivation have been identified as independent prognostic markers[23]. This data support a role of E2F-1 as tumor suppressor gene in lymphoma and strongly suggest that the RB1 and p53 pathways are important in the development of de novo diffuse large B cell lymphoma[25].

11. More recent studies implicate p73 in TCR activation-induced cell death (TCR-AICD), indicating that p73-alpha could play a tumor suppressor role in T-cells[26]. p73 has been found to be transcriptionally silenced in some lymphoblastic leukemias and lymphomas due to hypermethylation [27,28].

12. JunB, a transcriptional target of p73, which functions as a tumor suppressor in myeloid cells, has been shown to be induced by 13.4 fold in *Dnmt1*^-/- ^fibroblasts, indicating that DNMT can actively silence the expression of JunB when it is expressed[29] (figure 2). DNMT-1 is also shown to be a transcriptional target of c-Jun and Rb[30]. In addition, it has been shown that DNMT-1 specifically targets E2F1-1 element containing promoters and p73 promoter bears E2F1 responsive elements[31]. Evidently, DNMT1s were substantially overexpressed in leukemia cells in a leukemia type-and stage-specific manner. Up-regulated DNMTs may contribute to the pathogenesis of leukemia by inducing aberrant regional hypermethylation of tumor suppressor genes such as p73, JunB, p16, p57kip2, 14-3-3 and BRCA-1.

13. c-Jun has been shown to be activated in Hodgkin's lymphoma and anaplastic large-cell-lymphoma cell lines[32]. AP-1 complex largely contain c-JUN. NFkappa B is also constitutively activated in Hodgkin's lymphoma cells. It could be that overexpressed c-Jun(AP-1 complex) consistently silent p73 by turning on DNA methyl transfereases(DNMT-1) and thereby it could contribute for the development of lymphomas(figure 2). These data further suggest that p73 could behave like a tumor suppressor gene in Non-hodgkin lymphoma and in NK cell lymphoma's. p73 inactivation may be involved in the pathogenesis of both T- and B-ALLs, and that hypermethylation is the predominant mechanism of inactivation of the p73 gene in ALL. Together, it appears that overexpressed AP-1 could silence p73 in lymphocytes.

14. p73 is shown to be a transcriptional target of E2F-1[47]. Remarkably, E2F-1 null mice develop unusual spectrum of tumors, indicating that E2F-1 could behave like a tumor suppressor gene. Perhaps, E2F-1 may induce p73 expression to prevent tumorigenesis. Interestingly, E2F-1 null mice develop spelenomegaly and are resistant to apoptosis. Moreover, splenic T-cells isolated from p73-deficient mice are resistant to apoptosis induced by the ligation of the T-cell receptors, suggesting that p73 can also induce apoptosis in activated lymphocytes[26].

15. Overexpression of p73 has been shown to upregulate the expression of several DNA repair proteins including DNA-PK[33]. DNA-PKcs null mice demonstrate complete penetrance of thymic lymphoblastic lymphomas, strongly suggesting that DNAPK functions in mice as a T-cell tumour suppressor. Thus, down regulation of p73 could down regulate DNAPK expression and thereby predispose mice to thymic lymphoma formation.

16. CCAAT/enhancer binding proteins c/EBPs-alpha has been shown to be critical for both early myeloid commitment and terminal granulocyte differentiation. Both p73 and cEBP-alpha share PPXY motif, raising a possibility that both, in principle, could be regulated by a common WW domain containing co-activators such as TAZ or YAP to regulate myeloid or granulocite differentiation.

17. p73 has been shown to upregulate the expression of JEM-1, which is down regulated in acute promyelocytic leukemia[34].

18. NAD(P)H:quinone oxidoreductase 1 (NQO1) seems to stabilize both p53 and p73, inhibition of which reduces the levels of p53 and p73. Interestingly, disruption of the NQO1 gene in mice causes myelogenous hyperplasia[35], indicating the absence of p73 could possibly help develop myelogenous hyperplasia.

19. Promyeolocytic leukemia(PML) has been identified as one of the interacting proteins of p73 through the yeast two hybrid systems[36]. Further, PML has been shown to increase the stability and function of p73[37]. Retroviral expression of beta-catenin, plakoglobin, or PML suppressed the tumorigenicity of p53-negative human renal carcinoma cells, thus pointing to a novel antioncogenic response triggered by catenins that is mediated by the induction of PML.

## If indeed p73 is a tumor suppressor gene, then why p73 null mice failed to develop lymphoma?

### Explanation 1

**Figure 3:**
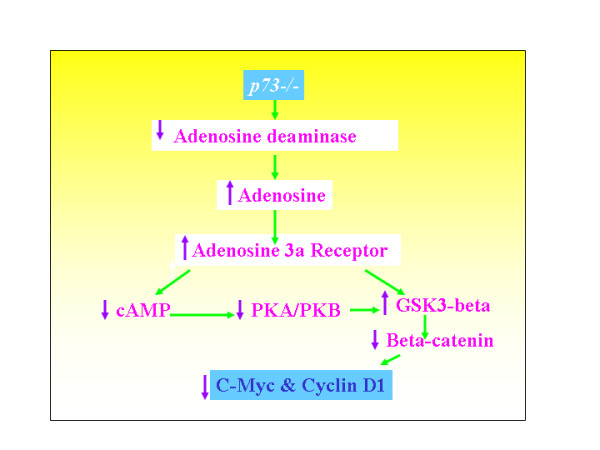
Increased adenosine concentration down regulates various proliferation regulating mechanisms.

It has been shown that adenosine deaminase (ADA) is a transcriptional target of p73 and p63[38,39]. Further, it has been shown that decreased expression of ADA could lead to increased levels of adenosine. In fact, it has been shown that ADA deficiency could lead to thymic apoptosis and defective TCR signaling. Hence, I hypothesize that p73 null mice will have high level of adenosine accumulated at various sites due to low expression of adenosine deaminase (ADA). If p73 and p63 are positive regulators of ADA, then ADA expression will be decreased in p73/p63 null mice that could result in increased adenosine level in p73/p63 null mice (figure 3). It has been shown that both adenosine and its agonists inhibit the growth of various tumor cell types such as melanoma, colon or prostate carcinoma and lymphoma [40-42]. This effect is specific and is exerted on tumor cells only. Increased adenosine level could:(a) Prevent initiation/formation of tumors by interfering with protein and RNA biosynthesis and thereby inhibit proliferation. In support of this hypothesis, the generalized suppression of growth is evident in p73 null mice. This could be due to increased extra and intra cellular levels of adenosine, as increased adenosine could inhibit the protein and RNA synthesis and thereby prevent tumor formation(figure 3). (b) Prevent lymphoma formation. (c) Potentiate the function of adenosine 3a receptor, which provides anti-proliferative signals[41]. Together, this explains why p73 null mice failed to develop lymphoma.

### Explanation 2

**Figure 4:**
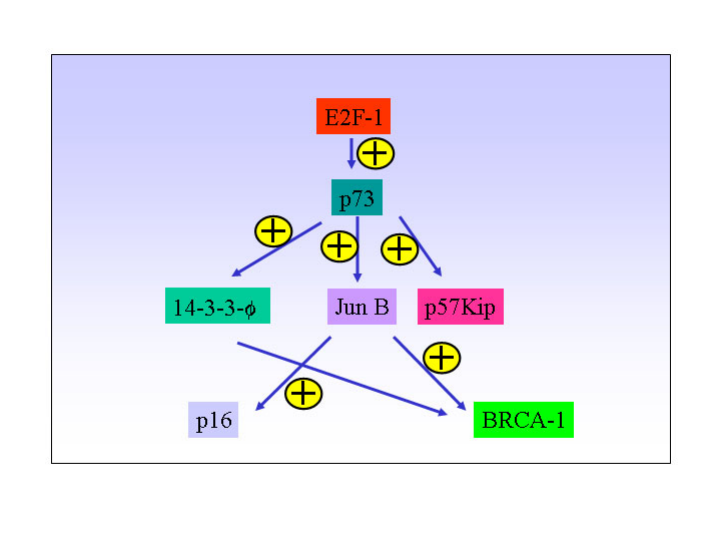
E2F-1-p73 tumor suppressor network.

What I intend to propose here is that p73 may not be a primary tumor suppressor gene, like its counter part p53, but it looks certain that it is a participant in the tumor suppressor network. The p73 promoter is strongly activated in cells expressing exogenous E2F1 and suppressed by exogenous Rb. E2F1 null mice develop various tumors including lymphoma. This might suggest that E2F1 could exert its anti-proliferative action via p73. In addition, E2F1 is capable of activating p19ARF and thereby it can increase the stability of p53 protein to control proliferation(figure 4). This is to say that, in the absence of p73, E2F could still control aberrant proliferation by turning on p19/ARF and thereby stabilizing p53. In the absence of p73 and p63, p53 is still capable of eliciting growth arrest – but not apoptosis – by transactivating p21 promoter[43]. This also explains why p73 null mice failed to develop lymphoma. The absence of E2F-1 affects both p53 stability and p73 expression and ARF/p19 expression. This explains why *E2F1*^-/- ^null mice develop tumors, unlike p73 null mice.

### Explanation 3

The tumor suppressor pathway in which p73 appears to be a participant involves E2F1, JunB, p16, p19, p57kip2 and BRCA1(figure 4). Each of which can elicit anti-proliferative effect on its own and in the absence of the other. In the absence of p73, Jun B transcription could still be transactivated by other factors. And JunB could still be capable of transactivating p16 expression and augment the function of BRCA1. Further, if p73 functions like its dictator, E2F-1, then we should have seen lymphoma in p73 null mice, but that is not the case. This can be explained if one considers the fact that E2F-1 tumor suppressor pathway or network, seems to control the expression and the stability of various tumor suppressor genes such as p19ARF, p53, JunB, p16, BRCA, p57Kip2 etc., in addition to p73(figure 4).

### Explanation 4

The facts that TA-p73 can induce apoptosis and it is hypermethylated in tumors of lymphoid origin indicate that this could function like a tumor suppressor gene. On the other hand, accumulating evidence suggest that delta-Np73 functions more like an oncogene [44,45]. Cancer is a genetic disease that arises through inactivation of tumor suppressor genes and activation of oncogenes. Removal of both oncogene and tumorsuppressor gene may not lead to spontaneous tumor formation. For example, this could be analogous to a situation where removal of both p27Kip2 (inhibitor of proliferation) and cyclin D1 (activator of proliferation) may not lead to tumor formation. Interestingly, p73 null mice lack both TA-p73 and deltaN-p73, perhaps this could be the reason why p73 null mice do not develop spontaneous tumors. In fact, it appears that p73 null fibroblasts proliferate slowly(Antonio et al., personal communication).

Considering these facts presented here one would not be surprised by the fact that why p73^-/- ^mice failed to develop lymphoma, unlike p53 null mice.

## Why do p73 mutations are infrequent in tumors?

1. p73 gene contains several alternative spliced forms, which vary in their ability to elicit growth suppressor response. This flexibility in function will be added factor for modulation, rather than complete inactivation. Thus, just by varying the expression of p73 splicing products(TAp73 Vs DN-p73) it can be finely regulated.

2. p73 promoter is rich in CpG rich islands, unlike p53 promoter. Thus, p73 is subject to methylation mechanisms mediated inactivation rather than point mutations.

3. p73 appears to co-operate with several proto-oncogenes such as, ras, E2F-1, cyclins (A-E), BCR-abl, and myc, therefore, it could be less prone to selection pressure for point mutations unlike p53. Both p53 and p73 seem to share upstream components especially in response to DNA damaging drugs. p53 is mutated or its upstream/downstream component is altered in 80% of cancer cells. If upstream component is altered then it may not be able to activate p73-mediated apoptotic pathway, so it may not perform its function effectively. MLH-1 is shown to be an upstream regulator of p73, unlike p53. MLH-1 is hypermethylated in a wide variety of tumors. Thus, in the absence of MLH-1 expression, p73 may not be activated and respond to DNA damage signals.

4. In-vitro studies support the view that E2F1 is overexpressed in various cell lines and it regulates proliferation, while *invivo *studies suggest that it is a tumor suppressor gene. Although knock-out studies in mice suggest that E2F-1 functions as a tumor suppressor, until now no mutation has been reported in human tumors. This could be due to the fact that complete inactivation of E2F1 function may be deleterious to the survival of normal/cancer cells, as E2F1 also regulates proliferation associated genes. So, it is prudent not to anticipate any mutations in E2F1 gene. Similarly, p73 being a direct transcriptional target of E2F-1, it could be part of the regulatory mechanisms that control proliferation signals as well. Moreover, p73 could act as an anti-apoptotic and a pro-apoptotic protein, depending upon the cellular context and expression pattern of p73 isoforms. Thus, p73 mutations are infrequent.

**Figure 5:**
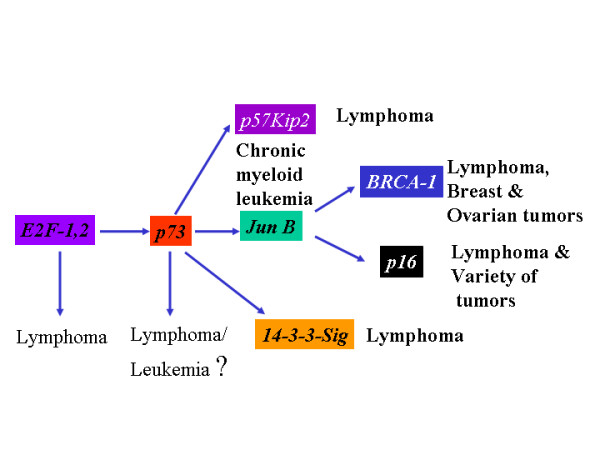
Knock out of each gene in E2F-1-p73 network of proteins result in lymphoma formation.

E2F1-deficient mice have been shown to develop various tumors including reproductive tract sarcomas, lymphoma and lung adenocarcinoma, suggesting that it functions as a tumor suppressor gene[46]. Subsequently, it has been shown that JunB, a negative regulator of proliferation, is a potential transcriptional target of the tumor suppressor p53 homolog, p73 [9,47]. Furthermore, p73 has been shown to be a transcriptional target of the tumor suppressor gene, E2F1[48] (figure 5). Wild-type mouse embryonic fibroblasts showed 77% apoptosis after forced E2F1 expression, both *p53*^-/- ^MEFs(expressing wild-type p73) and *p73*^-/- ^MEFs (expressing wild-type p53), showed reduced apoptotic cell death after forced E2F1 expression with 12% and 15% respectively[48]. The inability of *p73*^-/- ^mouse embryonic fibroblasts cells to undergo, despite the presence of wtp53, apoptosis indicate that synergistic but independent activation signal stemming from TAp73 that co-operate with p53 to induce E2F1-triggered cell death[48]. These findings strongly suggest that E2F1 could exert its tumor suppressor function, at least in part, via p73. In support of this notion, Zhu JW et al., [49] showed that *E2F1*^-/-^*E2F2*^+/- ^or *E2F1*^-/+^*E2F2*^-/- ^mice developed various tumors including T/B-cell-lymphomas, lung tumors and myeloid hyperplasia/leukemia. Accordingly, JunB – a transcriptional target of p73 – null mice develop chronic myeloid leukemia(CML) like disease ref. 12(figure 5), suggesting that JunB functions as p73's downstream target and it functions as a tumor suppressor gene in mice. In line with this data, it has further been shown that JunB expression is silenced in CML by hypermethylation[50] and it functions as a negative regulator of B-lymphoid proliferation[51] However, on the contrary to the expectation, p73 null mice failed to develop spontaneous lymphoma formation even after two years[52]. By contrast, p73 is hypermethylated in various tumors. How can we explain this tumor paradox? It is plausible that spontaneous tumor formation in p73 null mice could be prevented by a molecule(s), such as adenosine, produced in its absence.
